# *TRAF1* Gene Polymorphism Correlates with the Titre of Gp210 Antibody in Patients with Primary Biliary Cirrhosis

**DOI:** 10.1155/2012/487521

**Published:** 2012-10-22

**Authors:** Agnieszka Kempinska-Podhorodecka, Zakera Shums, Michał Wasilewicz, Ewa Wunsch, Malgorzata Milkiewicz, Dimitrios P. Bogdanos, Gary L. Norman, Piotr Milkiewicz

**Affiliations:** ^1^Medical Biology Laboratory, Department of Laboratory Diagnostics and Molecular Medicine, Pomeranian Medical University, 70-111 Szczecin, Poland; ^2^INOVA Diagnostics, San Diego, CA 92131-1638, USA; ^3^Liver Unit, Pomeranian Medical University, 70-111 Szczecin, Poland; ^4^Institute of Liver Studies, School of Medicine, King's College London, London SE5 9RS, UK

## Abstract

*Background*. Polymorphisms of *TRAF1* (Tumor necrosis factor receptor-associated factor 1) are associated with rheumatoid arthritis (RA). Whether *TRAF1* polymorphisms confer increased risk for primary biliary cirrhosis (PBC), an autoimmune liver disease which can co-exist with RA, is unknown. *Aim of the Study*. To assess the frequency of the RA-conferring susceptibility *TRAF1* polymorphisms rs3761847 and rs2900180 in a cohort of PBC patients. The association of *TRAF1* polymorphisms with clinical features and autoantibody markers was also analyzed. *Methods*. We studied 179 PBC patients and 300 controls. Samples were genotyped for *TRAF1* gene polymorphisms by real-time PCR. Autoantibodies were tested by ELISA. *Results*. The frequency of rs3761847 and rs2900180 polymorphisms did not differ between patients and controls. Laboratory or clinical features were not associated with specific polymorphisms. Gp210 autoantibody titres were conspicuously higher among GG homozygotes of rs3761847 as compared with AA homozygotes (*P* = 0.02). In contrast, antichromatin titers were higher in AA compared to GG rs3761847 homozygotes (*P* = 0.04). Rheumatoid factor IgG titres were significantly higher in rs2900180 TT homozygotes than CC homozygotes (*P* = 0.02). *Conclusions*. *TRAF1* polymorphisms occur with the similar frequency in PBC patients and in the general population, but their presence is probably involved in the regulation of specific PBC-related autoantibodies.

## 1. Background

Primary biliary cirrhosis (PBC) is a chronic cholestatic liver disease leading to cirrhosis and eventually liver failure and predominantly affects middle-aged women [1, 2]. Highly-specific antimitochondrial antibodies (AMA), as well as PBC-specific antinuclear antibodies such as those against sp100 and gp210, are serological hallmarks of the disease [3–10]. Significant proportion of patients with PBC suffer from concomitant autoimmune conditions such as Sjögren's syndrome, Hashimoto's thyroiditis, or systemic sclerosis (SS) [2, 11–15]. Several patients with PBC are found positive for IgG rheumatoid factor (RF), and up to 5% of patients with PBC may also have rheumatoid arthritis (RA) [16–18]. 

Although the etiology of PBC remains elusive, genetic factors are known to contribute to its pathogenesis [19–21]. Several studies in the past postulated major histocompatibility complex (MHC) class II polymorphisms as expressing the strongest association, but consensus has been reached mainly for genes exerting a reduced risk of developing PBC [22]. More recently, four genome wide association studies (GWAS) which included North American, Italian, predominantly North American, and British cohorts, respectively, identified a number of genes in non-HLA loci associated with PBC and highlighted the role of the IL-12 and tumor necrosis factor (TNF) signaling pathways in the pathogenesis of PBC [23–26].

Polymorphisms of genes involved in TNF-receptor signaling such as TNF receptor-associated factor 1 (*TRAF1*) have been associated with RA [27] and other autoimmune conditions including systemic lupus erythematosus (SLE) [28], but not with giant cell arteritis [29]. *TRAF1* gene encodes an important protein which acts as a mediator of the TNF and CD40 transduction pathways [30, 31]. The presence of *TRAF1* polymorphisms seems to affect the natural history of RA, increasing the risk of erosions [32]. Their effect on mortality in RA remains controversial [33, 34]. 

The association of *TRAF1* polymorphisms with the risk of developing PBC has not been studied. In the present study, we assessed the prevalence of the *TRAF1* polymorphisms rs3761847 and rs2900180, both identified by a large GWAS as genetic risk factors for RA [27], in a homogenous cohort of Caucasian patients with PBC. As *TRAF1* is a potent immune modulator, we postulated that the presence of *TRAF1* polymorphisms may predispose to a distinctive autoantibody profile and performed a comprehensive analysis of PBC-specific and nonspecific autoantibodies detected in our patients with PBC with the presence of *TRAF1* polymorphisms.

## 2. Methods

### 2.1. Patients

A group of 179 patients with PBC were analyzed. All patients met the criteria for the diagnosis of PBC recently introduced by EASL guidelines, according to which PBC can be diagnosed if at least 2 out of the following 3 criteria are fulfilled: elevation of alkaline phosphatase, typical liver histology, and AMA seropositivity [35]. In 132 (74%) patients, the diagnosis of PBC was confirmed by a liver biopsy, and in 46 (35%) of these patients histological assessment showed liver cirrhosis. Patients with clear clinical and imaging features of liver cirrhosis were not subjected to liver biopsy on ethical grounds. In total, 61 (34%) patients had histological, clinical, and imaging features typical for liver cirrhosis. Demographic and laboratory data on analyzed subjects are summarized in Table 1. A cohort of 300 blood donors from the National Blood Services comprised a control group. Appropriate informed consent was obtained from each patient and blood donors included in the study. The study protocol was approved by the ethics committee of Pomeranian Medical University and conforms to the ethical guidelines of the 1975 Declaration of Helsinki (6th revision, 2008).

## 3. Analysis of Autoantibodies

Autoantibody tests were performed at one site (INOVA Diagnostics, San Diego, CA, USA) on blinded serum specimens. A total of twelve autoantibody specificities (M2 EP (MIT3) IgG, gp210 IgG, sp100 IgG, chromatin IgG, centromere IgG, f-actin IgG, Scl-70 IgG, Jo-1 IgG, RNA polymerase III IgG, Ro52 IgG, CCP 3.0 IgG, and RF IgG) were analyzed by QUANTA Lite ELISA (INOVA Diagnostics, San Diego, CA, USA) as described elsewhere [4]. All QUANTA Lite ELISA tests were run and interpreted according to the manufacturer's instructions and are cleared for “*in vitro* diagnostic use” by United States Food and Drug Administration (FDA). Detailed performance data and instructions for the QUANTA Lite ELISA tests can be found online (http://www.inovadx.com). 

## 4. *TRAF1* Genotyping

DNA from peripheral blood mononuclear cells was isolated using the DNeasy Blood & Tissue Kit (Qiagen). Oligonucleotide primers and TaqMan probes for two *TRAF1* polymorphisms (rs2900180 and rs3761847) were designed and synthesized by Applied Biosystems (assay ID: C_15849116_10 and C_2783640_10, resp.). The fluorescence data were analyzed with allelic discrimination 7500 software v.2.0.2.

## 5. Statistical Analysis

Data are shown as mean and standard error. All statistical analyses (chi square, odds ratios, confidence intervals) were performed using StatView software (Carry, NC, USA). The genotype and allelic frequencies were compared between patients and controls using Fisher's PLSD test. The analysis of genotype frequency within PBC patients with regards to the analyzed factors was performed using Fisher's PLSD test. *P *value < 0.05 was considered to be statistically significant. 

## 6. Results

A summary of the obtained data is provided in Tables 2 and 3. No significant difference in genotype frequencies between patients with PBC and healthy controls was seen. The presence of these polymorphisms did not correlate with clinical features such as gender, age at presentation, pruritus, or cirrhosis at presentation (data not shown). Also they did not correlate with liver biochemistry at the diagnosis (Table 3). With regards to the rs3761847 polymorphism, analysis of autoantibodies revealed that the titres of gp210 were significantly higher among GG homozygotes as compared with AA homozygotes (42.6 ± 14.7 versus 10.8 ± 4.2; *P *= 0.02) and at the same time AA homozygoity was associated with higher titres of antichromatin autoantibodies (11.8 ± 2.8 versus 5.2 ± 1.2; AA versus GG *P *= 0.04). The analysis of the second polymorphism (rs 2900180) showed that TT homozygotes demonstrated significantly higher titres of rheumatoid factor IgG than CC homozygotes (16.7 ± 9.7 versus 7.6 ± 0.5, resp., *P *= 0.02). No statistically significant difference was found in terms of other autoantibodies. These data are summarized in Tables 4 and 5. 

## 7. Discussion

The *TRAF1* gene encodes a TNF receptor-associated factor 1, belonging to the TNF receptor (TNFR) associated factor (TRAF) protein family [36]. These proteins are responsible for mediation of signaling from various receptors of the TNFR superfamily. *TRAF1* together with *TRAF2* form a heterodimeric protein complex which is required for TNF-alpha-stimulated activation of MAPK8/JNK and NF-kappaB [37, 38]. This complex also interacts with proteins responsible for inhibition of apoptosis, affecting the antiapoptotic signals from TNFRs [39]. TNF*α* has been found to play a critical role in the pathogenesis of various autoimmune conditions including RA and PBC [40]. 

Various polymorphisms of *TRAF1*/C5 have been studied and were found to occur more commonly in patients with RA of different origin [41–44]. In addition to their higher prevalence in patients with RA, *TRAF1*/C5 polymorphisms also seem to affect the natural history of the disease [45–47]. They have also been linked with SLE in some populations [48, 49], juvenile idiopathic arthritis [50] and alopecia areata [51], but not with giant cell arteritis [29] and pemphigus [52]. 

*TRAF1* SNPs have never been investigated in PBC which is a chronic autoimmune liver condition. Since pathways involving TNF and IL-12 have been described [53–55] in the pathogenesis of PBC, seeking a potential relationship between *TRAF1* SNPs and PBC could be of interest. Additionally, PBC is associated with various autoimmune conditions including Sjögren's syndrome and systemic sclerosis, and the mechanisms responsible for this cooccurrence are the focus of ongoing research [56]. RA is found in up to 5% of patients with PBC, but the literature surrounding this association is scarce. 

For this study, we selected two polymorphisms originally reported to confer increased risk for RA in GWAS [27]. The frequencies of the SNPs that we studied are comparable to those noted previously in European and other populations (displayed in http://www.ncbi.nlm.nih.gov/SNP/snp_ref.cgi?searchType=&rs=rs3761847 and http://www.ncbi.nlm.nih.gov/SNP/snp_ref.cgi?searchType=adhoc_search&type=rs&rs=rs2900180). They were also not significantly different from the ones seen in patients with PBC, suggesting that the *TRAF1* locus does not confer risk to PBC. This may also explain why RA is not highly prevalent in patients with PBC compared to other extrahepatic autoimmune manifestations such as sicca syndrome and Hashimoto's thyroiditis. These findings support the view that genes other than *TRAF1* which are related to the homeostasis of TNF*α* could be involved in the pathogenesis of PBC. Indeed, a recent GWAS by Mells et al. reported that 1q31 (*DENND1B*), 14q32 (*TNFAIP2*), and 12p13 (*TNFRSF1A*) confer susceptibility to PBC. These three loci relate to genes involved in TNF signaling pathways. Of those, TNFRSF1A belongs to the TNFR family, which also contains TNFRSF1B. Of interest, TNFRSF1A appears to interact with *TRAF2* but not with *TRAF1* [57–59]. Our analysis has found that the GG homozygotes had significantly higher titres of gp210 autoantibodies compared to AA homozygotes. Antibodies against the nuclear complex gp210 antigen are highly-specific for PBC [6, 9, 60] and their presence is strongly associated with a more rapid progression of PBC and worse outcome [61–64]. The fact that the presence of this polymorphism is not associated with other PBC-specific antinuclear antibodies, such as those against the sp100 nuclear body antigen, further underlines the unique association between *TRAF1* and gp210 autoantibody development. The mechanism that could explain this association needs to be explored. *TRAF1* has an inhibitory role in antigen-induced apoptosis of CD8+ T lymphocytes. It remains to be seen whether such an antiapoptotic role involves gp210-specific autoreactive lymphocytes present in PBC patients [65]. As *TRAF1* is also a negative regulator of TNF-receptor signalling, it may regulate the induction a cytokine milieu that promotes the persistence of gp210-specific autoreactive lymphocytes and the T-cell dependent production of antibodies against this nuclear pore complex protein. These speculations may serve as working hypotheses for future studies. However, our data need to be interpreted with caution, as there is no solid evidence to support the view that anti-gp210 seropositivity *per se* is a negative prognostic factor in patients with PBC. Nevertheless, the idea to correlate polymorphisms of immunoregulatory genes with humoral autoimmunity markers is not new, as previous researchers have attempted the same in diseases such as type 1 diabetes mellitus, systemic lupus erythematosus, and indeed RA [66–68]. In doing that, several studies identified relationships between HLA and non-HLA polymorphisms and the presence or the titres of disease-related autoantibodies. To date, no serious attempts have been made to correlate *TRAF1* polymorphisms and specific autoantibodies in autoimmune diseases such as RA and SLE. 

Our study is the first to assess the presence of these polymorphisms with the titres of twelve different autoantibodies, including not only closely-related and clinically significant autoantibody specificities but also others not immediately relevant to the diagnosis or prognosis of PBC. Hence, we showed that antichromatin autoantibodies occurred in significantly higher titres in AA homozygotes compared to GG homozygotes of rs3761847. The significance of antichromatin antibodies has not yet been studied in great detail in patients with PBC. In patients with autoimmune hepatitis, however, the presence of antichromatin antibodies is associated with an active disease and increased risk of relapse after steroid withdrawal [69, 70]. It has been speculated that they may define a subgroup of patients with AIH with worse outcome [71]. We also observed significantly higher titres of RF IgG in TT homozygotes of rs2900180. Again, a direct interpretation of this finding is difficult to be made. Rheumatoid factor is related to more aggressive articular destruction in patients with RA. RF-IgG can occur in 16–70% of patients with PBC, but its relevance in the natural history of this condition has not been studied. Further studies should define the association of these autoantibodies and *TRAF1* polymorphisms in patients with PBC. Our study has moved this process one step forward. 

## Figures and Tables

**Table 1:** Main demographic and laboratory data of 179 patients with primary biliary cirrhosis.

Feature	PBC (*n* = 179)
Age (median; range)	56 (22–80)
Gender (M/F)	21/158
Biopsy confirmed cirrhosis (Y/N)	46/86
AMA (pos/neg)	155/24
ALT (median; range), IU/L (*N*: 3–30)	84 (10–727)
ALP (median; range ), IU/L (*N*: 40–120)	323 (37–2264)
GGT (median; range), IU/L (*N*: 3–30)	302 (11–2608)
Bilirubin (median; range), mg/dL (*N*: 0.2–1.0)	3.0 (0.2–40.5)
Albumin (median; range), g/dL (*N*: 3.8–4.4)	3.6 (2.1–5.2)
INR (median; range) (*N*: 0.8–1.2)	1.0 (0.8–2.3)
Cholesterol (median; range ), mg/dL (*N* < 200)	238 (50–709)
Triglycerides (median; range), mg/dL (*N* < 150)	124 (42–334)

AMA: antimitochondrial antibody; ALT: alanine aminotransferase; ALP: alkaline phosphatase; GGT: gamma-glutamyl transpeptidase; INR: international normalized ratio.

**Table 2:** Distribution of *TRAF* polymorphisms (rs3761847 and rs2900180) in patients with primary biliary cirrhosis (PBC) and controls.

SNP	Allele/genotype	PBC (%) (*n* = 179)	Controls (%) (*n* = 300)	*χ* ^2^	*P* (Fisher exact)	OR
rs3761847	*A/G *	210/148	356/244	0.07	0.84	1.04 [0.79–1.35]
	*AA *	64 (35.8)	99 (33)	0.38	0.55	1.13 [0.77–1.67]
	*AG *	82 (45.8)	158 (52.7)	2.12	0.16	0.76 [0.52–1.10]
	*GG *	33 (18.4)	43 (14.3)	1.41	0.25	1.35 [0.82–2.22]

rs2900180	*C/T *	114/244	185/415	0.13	0.72	1.05 [0.79–1.39]
	*CC *	84 (46.9)	144(48)	0.052	0.85	0.96 [0.66–1.39]
	*CT *	76 (42.5)	127 (42.3)	0.001	>0.99	1.02 [0.70–1.49]
	*TT *	19 (10.6)	29 (9.7)	0.11	0.75	1.11 [0.60–2.04]

**Table:** (a) rs3761847

Genotype	*AA *	*GG *	*P*
AST (IU/mL)	94.9 ± 18.1	67.5 ± 7.4	0.30
ALT (IU/mL)	91.5 ± 15.7	57.0 ± 5.3	0.16
AP (IU/mL)	342.1 ± 47.6	303.3 ± 36.8	0.58
GGT (IU/mL)	299.5 ± 45.4	367.1 ± 64.9	0.87
Bilirubin (mg/dL)	3.9 ± 0.9	3.1 ± 1.1	0.58
Albumin (g/dL)	3.9 ± 0.1	3.9 ± 0.1	0.83
INR	1.1 ± 0.1	1.1 ± 0.1	0.60
Cholesterol (mg/dL)	237.7 ± 16.9	225.7 ± 15.4	0.67

**Table:** (b) rs2900180

Genotype	*CC *	*TT *	*P *
AST (IU/mL)	77.9 ± 12.2	71.8 ± 10.7	0.85
ALT (IU/mL)	76.1 ± 9.3	66.2 ± 7.8	0.74
AP (IU/mL)	343.5 ± 38.4	332.1 ± 56.4	0.89
GGT (IU/mL)	314.6 ± 40.3	355.8 ± 109.6	0.67
Bilirubin (mg/dL)	3.5 ± 0.8	3.3 ± 1.7	0.88
Albumin (g/dL)	3.8 ± 0.1	3.7 ± 0.2	0.54
INR	1.1 ± 0.1	1.2 ± 0.1	0.33
Cholesterol (mg/dL)	232.1 ± 13.5	227.6 ± 21.7	0.89

Abbreviations: AST: aspartate aminotransferase; ALT: alanine aminotransferase; AP: alkaline phosphatase; GGT: gamma-glutamyl transpeptidase; INR: international normalized ratio.

**Table 4:** Autoantibody data with regards to rs3761847 *TRAF1* polymorphism.

Autoantibodies	Genotype *AA *	Genotype *GG *	*P*
AMA	99.8 ± 8.0	111.4 ± 10.9	0.38
gp210	10.8 ± 4.2	42.6 ± 14.7	0.02
sp100	31.7 ± 7.8	42.2 ± 13.5	0.42
Actin	23.7 ± 3.3	17.2 ± 2.6	0.20
Centromere	20.7 ± 5.7	14.1 ± 6.1	0.46
Chromatin	11.8 ± 2.8	5.2 ± 1.2	0.04
RFIgG	7.4 ± 0.7	13.8 ± 6.4	0.06
CCP3	16.6 ± 6.2	23.2 ± 11.1	0.45
Scl-70	4.3 ± 0.4	3.5 ± 0.3	0.25
Jo-1	4.7 ± 0.9	3.6 ± 0.5	0.81
RNA-POLIII	6.7 ± 1.2	4.9 ± 2.3	0.43
Ro52	25.7 ± 5.5	33.4 ± 8.3	0.49

AMA: antimitochondrial antibody; RFIgG: rheumatoid factor IgG; RNA-POLIII: RNA polymerase III.

**Table 5:** Autoantibody titers in patients subgrouped according to rs2900180 *TRAF1* polymorphism.

Autoantibodies	Genotype *CC *	Genotype *TT *	*P*
AMA	97.3 ± 7.1	108.1 ± 13.4	0.48
gp210	18.6 ± 5.7	35.5 ± 18.6	0.29
sp100	33.5 ± 6.7	24.2 ± 10.9	0.53
Actin	20.9 ± 2.5	15.6 ± 2.3	0.36
Centromere	17.4 ± 4.4	13.6 ± 8.4	0.72
Chromatin	0.9 ± 2.2	4.6 ± 1.1	0.18
RFIgG	7.6 ± 0.5	16.7 ± 9.7	0.02
CCP3	14.7 ± 4.8	31.2 ± 16.8	0.09
Scl-70	4.3 ± 0.4	3.4 ± 0.4	0.25
Jo-1	4.9 ± 0.8	3.6 ± 0.7	0.81
RNA-POLIII	6.4 ± 1.0	6.4 ± 3.9	0.97
Ro52	28.0 ± 5.2	42.2 ± 13.2	0.28
